# Effect of TOC Concentration of Humic Substances as an Electron Shuttle on Redox Functional Groups Stimulating Microbial Cr(VI) Reduction

**DOI:** 10.3390/ijerph19052600

**Published:** 2022-02-24

**Authors:** Yi Zhou, Jingtao Duan, Jie Jiang, Zhen Yang

**Affiliations:** 1 College of Environmental Science and Engineering, Beijing Forestry University, Beijing 100083, China; shjzhouyi@bjfu.edu.cn (Y.Z.); jingtaoduan@u.nus.edu (J.D.); 2 College of Urban and Environmental Science, Peking University, Beijing 100871, China

**Keywords:** humic substances, microbial Cr(VI) reduction, total organic carbon, electron shuttle, redox-active functional groups

## Abstract

Humic substances as an electron shuttle play an essential role in the biogeochemistry processes. However, the influence of total organic carbon (TOC) concentrations of humic substances on microbial Cr(VI) reduction remains unclear. In this study, the rates and extents of Cr(VI) reduction by *Shewanella oneidensis* MR-1 in the presence of Leonardite humic acids (LHA) and Pahokee peat humic acids (PPHA) with different TOC concentrations were evaluated. We found that the enhanced reduction in Cr(VI) was associated with TOC concentrations of 2.5–50 mg C/L of HA samples. The result shows that HA as an electron shuttle impacted both rates and extents of microbial Cr (VI) reduction, which delivered differently in terms of low TOC concentration range of 2.5 to 15 mg C/L and high concentration range of 15–50 mg C/L. The rates of Cr(VI) reduction significantly enhanced in the low TOC concentration range of HA compared to a high concentration range. The highest acceleration rate of Cr(VI) reduction was achieved at 15 mg C/L of HA. The quinone-like fluorophore was responsible for the main redox-active functional groups of HA by the three-dimensional excitation-emission spectroscopy. The fluorescence intensity of quinone-like fluorophore of HA in the low TOC concentration range was positively correlated with its acceleration coefficient, corresponding to the highest microbial Cr(VI) reduction rate obtained in 15 mg C/L of HA. These findings highlighted the effect of the TOC concentration of HA on microbial Cr(VI) reduction processes. It emphasized that the low TOC concentration of HA contributed to the high rates of Cr(VI) reduction, which is critical for better understanding the fate of Cr(VI) and evaluating the effectiveness of Cr(VI) restoration strategies in the future.

## 1. Introduction

Chromium, as one of the most widely used natural metal elements, is extensively released into the environment due to various industrial activities [1,2]. Two of the most stable oxidation forms of Cr, trivalent chromium (Cr(III)) and hexavalent chromium (Cr(VI)), are abundant in diverse environments [3]. Cr(VI) is more soluble, mutagenic, and toxic than Cr(III), which has aroused increasing public concern [4,5]. Biotransformation of Cr(VI) has thereby appeared and rapidly developed [6,7,8] as it is environment-friendly and cost-effective [9,10,11,12].

Humic substances are natural organic matter with abundant functional groups and it has been demonstrated to significantly influence transformation and transport of redox-sensitive contaminants such as Cr(VI), through sorption [13,14,15,16] and reduction [17,18,19], respectively. Humic substances are able to function as an electron shuttle that first accepts electrons from iron-reducing bacteria and then reduces humic substances and donates electrons to extracellular electron acceptors to stimulate extracellular electron transfer processes involved in microbial reduction in redox-active compounds such as Fe(III) hydroxides [20,21,22], Cr [23,24] and As [25,26]. The electron transfer capacities of humic substances are highly dependent on the redox-active functional groups (RAFGs) [27] that are generally associated with the supermolecule structure of humic substances [28,29] and total organic carbon (TOC) concentrations [30,31]. For instance, the total carbon contents of agricultural soils in Europe range from 0.4% to 46.0% [32]. The TOC contents of paddy soils are approximately 1.5% [33] in several Chinese provinces, and humus organic carbon occupy up to 80% of the soil organic carbon fraction in paddy soils [34]. In addition, the total carbon content in peat soil is generally higher than in agricultural soil in about 16.8–52.5% [35]. The TOC contents of agricultural peat soils in southeast France are 25.8–427.8 g/kg, of which humic carbon accounts for about 80% of TOC and humic acid is about 10% of the TOC content [36]. As for coal mining soils, the TOC contents are 18–35 g/kg in eastern India [37]. TOC contents of humic substances have been shown to be of particular importance for biotransformation of redox-active elements in environments, however, the effect of different TOC concentrations of humic substances as an electron shuttle on microbial Cr(VI) reduction remains unclear.

Fluorescence spectroscopy is typically used to characterize and distinguish the fluorophore structure in humic substances. The typical fluorophores in humic substances include humic-like fluorophores and protein-like fluorophores. Characteristic fluorescence properties includes excitation wavelength/emission wavelength data and fluorescence intensities. The fluorescent structure characteristics of quinone-like compounds are responsible for the humic-like fluorophore characteristics of humic substances. Quinone/hydroquinone moieties are considered to be the main RAFGs during the electron transport processes [24,38]. Anthraquinone-2,6-disulfonate (AQDS) is a well-known quinonal moiety model chemical. It has been demonstrated that AQDS functioning as an electron acceptor is capable of stimulating and promoting reduction in trivalent iron oxide [20,39], and hexavalent chromium [24,40]. The electron transfer capacities of humic substances are related to the characteristics of quinone-like fluorophore, which promotes the significant differences in electron transfer capacities of humic substances within different TOC concentrations.

To this end, the study first aims to evaluate the extents and rates of microbial Cr(VI) reduction by *Shewanella oneidensis* MR-1 with different TOC concentrations of humic acids (HA). We used *Shewanella oneidensis* MR-1 as a model Fe(III)-reducing bacterium which is ubiquitous in freshwater, marine, soil and sedimentary environments. We selected two standard HA including Leonardite humic acids (LHA) and Pahokee peat humic acids (PPHA) with the low TOC range of 0–15 mg C/L and high TOC range of 20–50 mg C/L. Afterwards, an acceleration coefficient was developed to clarify the relationship between the rates of Cr(VI) reduction and different TOC concentrations of HA. The second aim of this study was to clarify the RAFGs distribution of PPHA and LHA with different TOC concentrations by the three-dimensional excitation-emission spectroscopy (3DEEM), and finally to exhibit its effects of TOC relating to RAFGs in HA on microbial Cr(VI) reduction processes.

## 2. Materials and Methods

### 2.1. Preparation of HA Solution

LHA and PPHA were purchased from the International Humic Substances Society (IHSS). Leonardite is produced by the natural oxidation of exposed lignite obtained from the Gascoyne Mine in Bowman County. The Pahokee peat is a typical agricultural peat soil of the Florida Everglades. The content of humic substances in soil can reach tens to hundreds of mg C/L, while the content in groundwater varies from 0.1 mg C/L to tens of mg C/L [41]. Therefore, used HA concentration in this study was selected in the range of 2.5–50 mg C/L relating to concentration range of humic substances in natural environments. HA samples were completely dissolved in a sterilized phosphate buffer solution (PP buffer; 50 mM, pH 7.0) without non-soluble residue in the finalized solution. HA were gradient diluted to be a series of final TOC concentrations of 2.5, 5, 10, 15, 20, 25, 50 mg C/L. TOC concentrations of the HA samples were determined by TOC-Vcsn analyzer (SHIMADZU). Prepared HA samples were stored at 4 °C in the dark.

### 2.2. Bacterial Strain and Culture Conditions

The optical density of *Shewanella oneidensis* MR-1 was determined at 600 nm absorbance (OD_600_) by microplate reader (RT-6000, Rayto). *Shewanella oneidensis* MR-1 was cultured in Luria-Bertani (LB) medium at 30 °C for 16 h (OD_600_ > 0.9). The bacterial cells were collected by centrifugation at 8000 rpm for 10 min, then washed three times and re-suspended in the *Shewanella* basal medium (containing HEPES buffer solution, pH 7.2) to achieve final OD_600_ of 10 for following experiments.

### 2.3. Microbial Cr(VI) Reduction Mediated by HA

All experiments were conducted in a 5 mL tube containing 2 mL reaction medium including *Shewanella oneidensis* MR-1 (4 × 10^7^ cells/mL), K_2_Cr_2_O_7_ (Cr(VI) final concentration of 2 mM in reactor) as the terminal electron acceptor, HA with different TOC concentration range of 2.5–50 mg C/L as an electron shuttle, and lactate (20 mM) as the sole electron donor. The pH 5.0 in the reactor was adjusted by H_3_PO_4_ due to the fact that the Cr(VI) reduction process consumes H^+^ affecting solution pH [42,43,44]. The experiments were kept in shaking (200 rpm) in the oxygen-free glove box (100% N_2_ gas, ZDP02-B). Cr(VI) in the supernatant was quantified using a diphenyl carbazide assay [45]. All experimental data were obtained from the average of a duplicate set up.

### 2.4. 3DEEM Spectroscopy Analysis

HA samples in different TOC concentrations were measure in a standard 10 mm quartz cell using a spectrofluorometer (F7000) equipped with 1500 W Xe lamp (Ushio Inc. Japan). 3DEEM were generated at 23 ± 2 °C and at excitation (Ex) and emission (Em) slit intervals of 5.0 nm in each band-pass. Fluorescence spectra of HA samples with different TOC concentrations were collected under an Ex range from 300 to 500 at 5 nm intervals and an Em range from 400 to 600 at 5 nm intervals. LMWF fluorescence spectra were collected under an Ex range from 200 to 600 at 5 nm intervals and an Em range from 220 to 600 at 1 nm intervals. Ex and Em were then corrected using the phosphate buffer sample 3DEEM. Intensities were normalized to the area under the phosphate buffer to obtain relative fluorescence intensities.

## 3. Results

### 3.1. The Stimulated Extents and Rates of Microbial Cr(VI) Reduction by HA with Different TOC Concentrations

**Figure 1:**
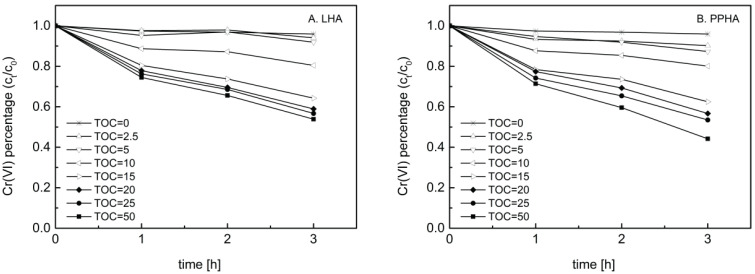
Influence of TOC concentrations of HA samples (2.5, 5, 10, 15, 20, 25, 50 mg C/L) on Cr(VI) reduction by *Shewanella oneidensis* MR-1. (**A**) LHA; (**B**) PPHA.

In order to explore the influence of HA with different concentrations on the microbial Cr(VI) reduction, we conducted microbial Cr(VI) reduction experiments by *Shewanella oneidensis* MR-1 in the absence and presence of LHA and PPHA with different TOC concentrations of 2.5, 5, 10, 15, 20, 25 and 50 mg C/L, respectively. The extents of microbial Cr(VI) reduction mediated by different TOC concentrations of LHA and PPHA was shown in Figure 1. 4.07% Cr(VI) and was reduced in the absence of HA at the end of incubation, while 5.95% Cr(VI) was reduced with addition of 2.5 mg C/L of LHA. Notably, the extent of Cr(VI) reduction greatly increased up to 35.74% and 46.11% with supplementation of 15 mg C/L and 50 mg C/L of LHA, respectively. For PPHA, we found the similar results as LHA, for both HA samples, up to 50% of extent of Cr(VI) reduction was observed by adding high TOC concentration range of 15–50 mg C/L of HA, whereas approximately 30% of Cr(VI) was reduced by amending with low TOC range of 2.5–15 mg C/L of HA.

**Figure 2:**
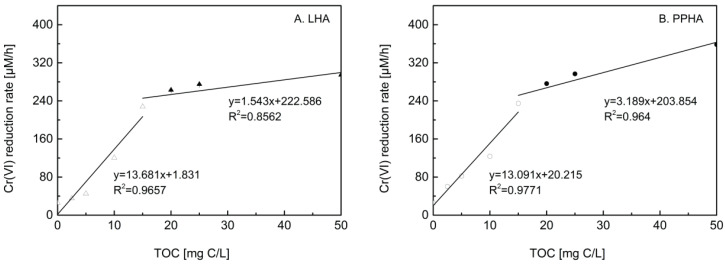
The relationship between TOC concentrations and rates of Cr(VI) reduction was fitted by a linear fitting slope K during different ranges of TOC concentrations. (**A**) LHA (△\▲); (**B**) PPHA (○\●). Hollow icons (△\○) represent low TOC concentrations of 2.5–15 mg C/L and solid icons (▲\●) represent high TOC concentrations of 20–50 mg C/L.

The Cr(VI) reduction rates were quantified under conditions of the different TOC concentrations of HA, which were further fitted based on the pseudo first-order reaction kinetic model (Figure 2) to figure out the detail acceleration effects of microbial Cr(VI) reduction. In the control experiment without adding HA (TOC = 0), the rate of Cr(VI) reduction was 25.6 μM/h. Compared to the control experiment (no HA addition), the rates of Cr(VI) reduction were increased from approximately 50 to 360 μM/h by adding HA samples in the TOC concentration range of 2.5–50 mg C/L for both LHA and PPHA. In addition, a higher TOC concentration in the range of 2.5–50 mg C/L in HA contributed to a faster rate of microbial Cr(VI) reduction. Notably, the rates of Cr(VI) reduction sharply increased 200 μM/h by adding TOC concentrations of HA from 0 to 15 mg C/L, compared to only about 100 μM/h of Cr(VI) reduction rate which was observed for TOC concentrations of HA increasing from 15 mg C/L to 50 mg C/L.

### 3.2. Characteristics of Functional Groups Distribution of Different TOC Concentrations of HA by 3DEEM

**Figure 3:**
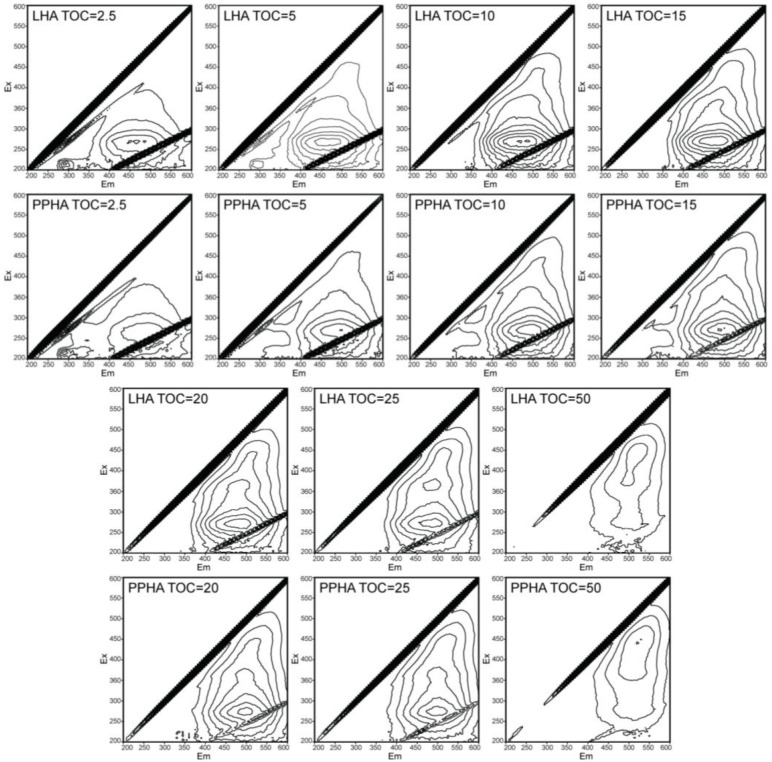
3DEEM fluorescence spectra of PPHA and LHA samples with different TOC concentrations.

3DEEM was employed to evaluate the characterizations of functional groups of humic substances at different TOC concentrations. The detail fluorescence information was shown in Figure 3 and Table 1. The fluorophore types of LHA and PPHA include protein-like fluorophores and humic-like fluorophores. Quinone-like fluorophore is the main composition of humic-like fluorophore produced by quinone π-π* and benzene π-π* transition (Ex/Em = 350–360/400–490 nm), which is responsible for electron transfer of HA. It was obvious that LHA and PPHA both had a great relative fluorescence intensity of humic-like flourophores (0.37–0.87 a.u.) compared to protein-like fluorophores (0.02–0.33 a.u.) within the whole TOC concentration range of 2.5–50 mg C/L. In addition, the quinone-like fluorophore accounted for a large proportion of humic-like fluorophores.

In high TOC concentration range of 20–50 mg C/L, intensities of quinone-like fluorophores were found gradually decreasing for both HA samples due to fluorescence-quenching occurrence, but a red-shifting in the peak positions appeared in high TOC concentration compared to in low TOC concentration range. For low TOC concentration range of HA increasing from 2.5 mg C/L to 15 mg C/L, intensities of quinone-like fluorophores were observed increasing along with the TOC concentration, and the red-shifting in peak positions of quinone-like fluorophores to a longer wavelength were also found.

## 4. Discussion

### 4.1. Effect of TOC Concentrations on HA Accelerating Microbial Cr(VI) Reduction

The low reduction extent of Cr(VI) at 2.5 mg C/L implied that 2.5 mg C/L of HA as an electron shuttle only minorly increased extent of Cr(VI) reduction compared to the control experiment. Besides, the result that a higher extent of Cr(VI) reduction has been observed with the addition of HA with high TOC concentrations is consistent with Mohamed’s [46] and Huang’s [47] studies that found that humic substances acting as an electron shuttle can transfer the electrons from microorganisms to Cr(VI). Similarly, AQDS promotes microbial reduction in chromate [39,48] and the reduction rate of Cr(VI) is positively correlated with AQDS concentrations [39,40], which supported the results in this study that both LHA and PPHA were capable of acting as an electron shuttle to stimulate microbial Cr(VI) reduction and the stimulation effect was related to the TOC concentrations of HA. In short, the extent of microbial reduction in Cr(VI) was steeply promoted in the high TOC concentration range compared to the low TOC concentration range.

The presence of HA improving the rate of microbial Cr(VI) reduction also indicated that HA could function as an electron shuttle to accelerate Cr(VI) reduction by MR-1. Notably, the difference of reduction rate varying with TOC concentration of HA suggested that there were dichotomy differences in the rates of microbial Cr(VI) reduction between TOC concentration range of 0–15 mg C/L and TOC concentration range of 15–50 mg C/L for both HA. Overall, HA as electron shuttle impacted both rates and extents of microbial Cr(VI) reduction and the effects were not linearly correlated with a whole TOC concentration range of HA.

To clarify the relationship between TOC concentrations and rates of Cr(VI) reduction, the linear fitting slope K of Cr(VI) reduction rates during different ranges of TOC concentrations was shown in Figure 2. For both HA, the K was up to 13 mmol_Cr(VI)_/h·g C_HA_ for the TOC concentration range of 0–15 mg C/L, whereas K dropped to 1–3 mmol_Cr(VI)_/h·g C_HA_ for the TOC concentration range of 15–50 mg C/L. The results present an obvious contradictory tendency at the turning point of 15 mg C/L of TOC concentration, indicating the higher Cr(VI) reduction rates obtained in the low TOC concentration range than in the high TOC concentration range. To rule out original rates of Cr(VI) reduction in the absence of HA, an acceleration coefficient (mmol_Cr(VI)_/h·g C_HA_) was developed to quantify the differences in reduction rates of Cr(VI) reduction among HA samples. The acceleration coefficients were normalized to difference in TOC concentrations between the corresponding TOC concentrations of HA and 0 (without addition of HA). The simplified acceleration coefficient is highly dependent on a specific TOC concentration of HA. The acceleration coefficients of LHA and PPHA at different TOC concentrations were shown in Table 2. For both HA, the highest acceleration coefficient was approximately 13 mmol_Cr(VI)_/h·g C_HA_ at 15 mg C/L of TOC concentration, suggesting that 15 mg C/L of HA had the greatest acceleration effect on the rate of microbial Cr(VI) reduction, given that 15 mg C/L of HA samples is equivalent to approximately 25 g/kg of HA components in Leonardite and Pahokee peat soils, which are available to most of the actual coal mining soils and peatland soil (soil types of used HA sources). Our findings expect to offer a theoretical support for Cr(VI) transformation of Cr(VI) to Cr(III) which has lower toxicity and less environmental mobility in soils. The methods used in the study might guide Cr(VI) restoration of soils in the reported coal mining areas where up to 500 mg Cr/kg soil in both coal mining soils and agricultural soils [48,49] and the 18–35 g TOC/kg soil in coal mining soils [50] have been reported.

### 4.2. Relationship between Acceleration Capability and RAFGs for Different TOC Concentrations of Humic Substances

The types of functional groups and fluorescence intensity displayed in 3DEEM fluorescence spectra suggested that the quinone-like functional groups were the predominant RAFGs for different TOC concentrations of HA. The red-shifting in the high TOC concentration supported that the quinone-like groups were responsible for the redox reactions of HA samples, and produced higher levels of conjugated chromophores in the quinone-like groups than low TOC concentration range of 2.5–15 mg C/L, particularly. These complicated quinone-like fluorophores contributed to a high extent of Cr(VI) reduction mediated by high TOC concentration range of HA. Changes of fluorescence intensities and peak positions at low TOC concentration range showed that the amount of quinone-like functional groups increased, which gradually changed to high conjugation chromophores within the low TOC concentration range from 2.5 mg C/L to 15 mg C/L until the highest amount and most complicated quinone moieties at 15 mg C/L of HA. This formation of the large amount and complicated quinone moieties in 15 mg C/L may provide an explanation for the fastest rate of Cr(VI) reduction mediated by 15 mg C/L of HA.

**Figure 4:**
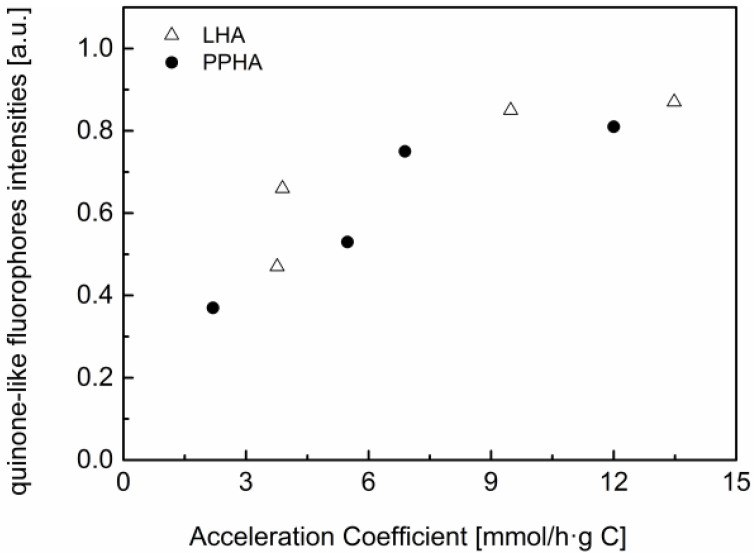
Relationship between the relative quinone-like fluorophores intensities of humic substances and their acceleration coefficients in the low TOC concentration range of 2.5–15 mg C/L.

In order to find out the effects of RAFGs in HA on microbial Cr(VI) reduction processes, a relationship was developed, particularly in the low TOC concentration range of 2.5–15 mg C/L, between the quinone-like fluorophores and its acceleration coefficients (Figure 4). For both LHA and PPHA, the relative fluorescence intensities of quinone-like fluorophores were positively correlated with their acceleration coefficients. HA has more of the amount of quinone moieties, which resulted in a higher acceleration coefficient. This relationship is exactly corresponding to the previous result that the obvious acceleration rates of microbial Cr(VI) reduction with increasing HA concentration range of 2.5–15 mg C/L until maximum rates of Cr(VI) reduction occurred in the presence of 15 mg C/L of HA.

## 5. Conclusions 

The extents of Cr(VI) reduction were promoted with increasing TOC concentrations of HA from 2.5 mg C/L to 50 mg C/L. We found that the rate of Cr(VI) reduction depended on the low or high TOC concentration range of HA. The rates of Cr(VI) reduction had significantly positive correlation with low concentration range of HA of 2.5 mg C/L to 15 mg C/L. Additionally, 15 mg C/L of HA displayed the highest acceleration rate of microbial Cr(VI) reduction. The quinone-like functional groups were the main RAFG of HA by 3DEEM spectroscopy. For both HA samples, the highest amount of quinone moieties at 15 mg C/L of HA led to the highest acceleration coefficient, which was responsible for the fastest rate of microbial Cr(VI) reduction. This study shed a new insight into how different TOC concentrations of HA impacts microbial reduction in Cr(VI) processes, particularly a different tendency was observed in reduction rates of Cr(VI) between low and high TOC concentration ranges of HA. Collectively, these findings are useful to better understand the factors controlling Cr reduction within varied carbon contents of soil environments. These laboratory-based findings can be expected to provide potential theoretical support for in-situ bioremediation in the Cr-contaminated coal mining soils and agricultural peatland soils.

## Figures and Tables

**Table 1:** Fluorescence peak positions and relative fluorescence intensities of PPHA and LHA with different TOC concentration.

Humic Substances Samples	TOC	Ex/Em ^a^	Intensity ^b^ [a.u.]	Peak Type	Transition Type
	[mg C/L]	[nm]			
LHA	2.5	275/305	0.31	protein-like	
		270/465	0.35	quinone-like	quinone π-π*
		355/410	0.12	quinone-like	benzene π-π*
	5	275/305	0.20	protein-like	
		270/480	0.49	quinone-like	quinone π-π*
		355/480	0.17	quinone-like	benzene π-π*
	10	275/305	0.10	protein-like	
		270/485	0.61	quinone-like	quinone π-π*
		355/480	0.24	quinone-like	benzene π-π*
	15	205/350	0.08	protein-like	
		270/485	0.59	quinone-like	quinone π-π*
		365/485	0.28	quinone-like	benzene π-π*
	20	210/365	0.08	protein-like	
		270/490	0.52	quinone-like	quinone π-π*
		365/485	0.29	quinone-like	benzene π-π*
	25	205/360	0.07	protein-like	
		270/490	0.44	quinone-like	quinone π-π*
		365/485	0.29	quinone-like	benzene π-π*
	50	205/355	0.04	protein-like	
		270/495	0.17	quinone-like	quinone π-π*
		455/495	0.21	quinone-like	benzene π-π*
PPHA	2.5	275/305	0.33	protein-like	
		270/490	0.26	quinone-like	quinone π-π*
		350/400	0.11	quinone-like	benzene π-π*
	5	275/305	0.22	protein-like	
		270/500	0.41	quinone-like	quinone π-π*
		360/415	0.12	quinone-like	benzene π-π*
	10	275/305	0.13	protein-like	
		270/500	0.53	quinone-like	quinone π-π*
		355/495	0.22	quinone-like	benzene π-π*
	15	220/350	0.08	protein-like	
		270/490	0.54	quinone-like	quinone π-π*
		365/495	0.27	quinone-like	benzene π-π*
	20	210/330	0.07	protein-like	
		275/495	0.50	quinone-like	quinone π-π*
		360/500	0.29	quinone-like	benzene π-π*
	25	215/345	0.06	protein-like	
		275/495	0.44	quinone-like	quinone π-π*
		365/500	0.29	quinone-like	benzene π-π*
	50	215/355	0.02	protein-like	
		280/500	0.17	quinone-like	quinone π-π*
		455/505	0.24	quinone-like	benzene π-π*

^a^ Ex/Em represented the excitation/emission wavelength, which was related to the position of the main fluorophore. ^b^ the fluorescence intensities here refer to the relative fluorescence intensities, which were the ratios of the fluorescence intensities at the peak positions to the fluorescence intensity of the PP buffer (50 mM, pH 7) at the same excitation wavelength when the emission wavelength is 355 nm: same as below.

**Table 2:** The calculated acceleration coefficients based on the rates of Cr(VI) reduction by LHA and PPHA samples at corresponding different TOC concentrations.

TOC [mg C/L]		2.5	5	10	15	20	25	50
Acceleration Coefficient ^a^ [mM_Cr(VI)_/h·g C_HA_]	LHA	3.76	3.89	9.48	13.48	11.86	9.97	5.38
	PPHA	2.19	5.48	6.89	12.90	11.08	9.68	6.07

^a^ $\mathrm{Acceleration}\;\mathrm{coefficient}\;=\frac{{\mathrm{reduction}\;\mathrm{rate}}_{a}{\;-\;\mathrm{reduction}\;\mathrm{rate}}_{0}}{{\mathrm{TOC}}_{a}-0}$.

## Data Availability

The experimental data used to support the findings of this study are included in the article. More detailed data are available from the corresponding author upon request.
